# Sex Differences in Characteristics and Outcomes in Elderly Heart Failure Patients With Preserved Ejection Fraction: A *Post-hoc* Analysis From TOPCAT

**DOI:** 10.3389/fcvm.2021.721850

**Published:** 2021-10-04

**Authors:** Jiaxing Sun, Shi Tai, Yanan Guo, Liang Tang, Hui Yang, Xuping Li, Zhenhua Xing, Liyao Fu, Shenghua Zhou

**Affiliations:** ^1^Department of Cardiovascular Medicine, The Second Xiangya Hospital of Central South University, Changsha, China; ^2^Department of Blood Transfusion, The Second Xiangya Hospital of Central South University, Changsha, China

**Keywords:** sex differences, HFpEF, baseline characteristics, mortality, HF-related hospitalization, elderly patients

## Abstract

**Introduction:** Although the impact of sex on patient outcomes for heart failure (HF) with preserved ejection fraction (HFpEF) has been reported, it is still unclear whether this impact is applicable for elderly patients with HFpEF. This study was conducted as a secondary analysis from a large randomized controlled trial—The Treatment of Preserved Cardiac Function Heart Failure with an Aldosterone Antagonist Trial (TOPCAT)—to evaluate the impact of sex differences on the baseline characteristics and outcomes of HFpEF patients who were older than 70 years.

**Methods:** Baseline characteristic of elderly patients were compared between men and women. Primary outcomes were cardiovascular (CV) mortality and HF-related hospitalization, whereas secondary outcomes were all-cause mortality and all-cause hospitalization. Cox regression models were used to determine the effect of sex differences on patient outcomes.

**Results:** A total of 1,619 patients were included in the study: 898 (55.5%) women and 721 (44.5%) men. Age was similar between women and men. Women had fewer comorbidities but worse cardiac function than men. The rate of primary outcomes was lower in women than in men (18.4 vs. 27.5%; *p* < 0.001), including rate of CV mortality (8.9 vs. 14.8%; *p* < 0.001) and HF-related hospitalization (13.4 vs. 18.2%; *p* = 0.008). All-cause mortality was also lower in women than in men (15.6 vs. 25.4%; *p* < 0.001). After adjustment for baseline characteristics, Cox regression analysis showed that female sex was a protective factor for CV mortality [hazard ratio (HR): 0.53; 95% confidence interval (CI): 0.40–0.73], HF-related hospitalization (HR: 0.71; 95% CI: 0.55–0.93), and all-cause mortality (HR: 0.59; 95% CI: 0.47–0.75). Although spironolactone significantly reduced the rate of all-cause mortality in women even after adjusting for baseline characteristics (HR: 0.68; 95% CI: 0.48–0.96; *p* = 0.028), no significant multivariate association was noted between sex and treatment effects (*p* = 0.190).

**Conclusion:** Among elderly patients with HFpEF, women had worse cardiac function but better survival and lower HF-related hospitalization rate than men.

**Clinical Trial Registration:** NCT00094302 (TOPCAT). Registered October 15, 2004, https://www.clinicaltrials.gov/ct2/show/NCT00094302.

## Introduction

The incidence and prevalence of heart failure (HF) with preserved ejection fraction (HFpEF) increase exponentially with advancing age (1). And clinical and echocardiographic characteristics, quality of life, and patient outcomes differ between young and old HFpEF patients (2). Most of the recently completed large randomized clinical trials on HFpEF did not specifically set an age limit for participants, and elderly patients whose clinical features, event rates, and response to treatments may be different from those of young patients are underrepresented in randomized clinical trials.

Sex differences exist in almost every facet of HF (both HF with reduced ejection fraction and HFpEF), including baseline characteristics, risk factors, pathophysiology, drug response, and patient outcomes (3–5). Community-based studies indicated that women are substantially different from men in terms of clinical features and event rates (6, 7). The Irbesartan in Heart Failure with Preserved Ejection Fraction (I-PRESERVE) trial demonstrated a lower mortality or hospitalization rate for both cardiovascular (CV) and non-CV diseases in women with HFpEF, suggesting a better overall prognosis in women (8). The Treatment of Preserved Cardiac Function Heart Failure with an Aldosterone Antagonist Trial (TOPCAT) reported no significant interactions between spironolactone and sex in terms of primary outcomes in a pre-specified subgroup analysis (9), whereas a secondary analysis of this trial, which was restricted to the Americans, has shown that women have a significantly decreased all-cause mortality rate associated with spironolactone (10); however, this was not observed in men, suggesting sex differences in patient outcomes. But information about sex differences on patient outcomes and spironolactone response in elderly patients with HFpEF is limited. To address this, our study conducted a *post-hoc* exploratory subgroup analysis in elderly patients with HFpEF from TOPCAT to determine sex differences in baseline characteristics, outcomes, and spironolactone response.

## Materials and Methods

### Study Design and Patients

For this *post-hoc* analysis, clinical data from TOPCAT were collected from the National Heart, Lung, and Blood Institute's Biological Specimen and Data Repository Information Coordinating Center (BioLINCC, Calverton, Maryland, USA). Patients who were diagnosed with symptomatic HF and left ventricular ejection fraction of ≥45% and were hospitalized for HF within 12 months prior to enrollment or had elevated natriuretic peptide levels (brain natriuretic peptide level (BNP) of ≥100 pg/ml or N-terminal pro-BNP level of ≥360 pg/ml) within 60 days prior to inclusion were eligible. The age of the patients had to be 50 years or above, with controlled blood pressure (systolic blood pressure of <140 mmHg or ≤160 mmHg if three or more drugs were used to control blood pressure) and serum potassium level of <5.0 mmol/L. Patients whose life expectancy was <3 years, estimated glomerular filtration rate was <30 ml/min/1.73 m^2^ of body surface area, or serum creatinine level was ≥2.5 mg/dl were excluded. The details can be found in the main study publications (9, 11). All included patients were randomly assigned to receive spironolactone or placebo treatment according to a double-blind design. For the purposes of our study, we selected 1,619 elderly patients (age ≥70 years) (12, 13) from TOPCAT to conduct a *post-hoc* secondary analysis.

### Definitions of Outcomes

The follow-up time was about 3.3 years. Primary outcomes were cardiovascular (CV) mortality and HF-related hospitalization, and secondary outcomes were all-cause mortality (CV and non-CV mortality) and all-cause hospitalization.

### Statistical Analysis

Descriptive statistical data were obtained for all variables of interest. Baseline clinical characteristics of patients were expressed as mean ± standard deviation for normally distributed continuous variables, median (interquartile range) for non-normally distributed continuous variables, and frequencies and percentages for categorical variables. Data were stratified by sex and treatment arms. Sex differences in outcomes were compared within the entire cohort, the placebo arm, and the spironolactone arm. All continuous variables were compared using the *t*-test or Mann–Whitney *U*-test, and categorical variables were compared using Fisher's exact test or the χ^2^ test. The Kaplan–Meier method was performed for time-to-event analysis. Associations between sex and patient outcomes were determined using univariate and multivariate Cox proportional hazards regression models. Adjusted variables included race, New York Heart Association (NYHA) class, myocardial infarction, atrial fibrillation, hypertension, dyslipidemia, chronic obstructive pulmonary disease, percutaneous coronary intervention, coronary artery bypass grafting, systolic blood pressure, diastolic blood pressure, heart rate, body mass index, baseline estimated glomerular filtration rate, and baseline potassium level. Stata/S.E. version 15.0 software (Stata Corp., College Station, TX, USA) was used for statistical analyses. Empower Stats was used to analyze sex–treatment interactions. Values of *p* < 0.05 were considered statistically significant.

## Results

### Baseline Characteristics According to Sex in the Elderly

Baseline characteristics of the overall cohort (*n* = 1,619) according to sex are summarized in Table 1. Of the 1,619 elderly patients, 898 (55.5%) were women, and 721 (44.5%) were men. All the patients were ≥70 years, and their mean age was similar between women and men. There were 533 (59.4%) women from the Americas (including the United States, Canada, Brazil, and Argentina) and 365 (40.6%) from Russia/Georgia. The baseline characteristics of each group are presented in Table 1. Women had fewer comorbidities than men: atrial fibrillation (41.4 vs. 47.4%; *p* = 0.016), myocardial infarction (18.7 vs. 30.9%; *p* < 0.001), coronary artery bypass grafting (7.6 vs. 22.3%; *p* < 0.001), percutaneous coronary intervention (12.5 vs. 18.9%; *p* < 0.001), dyslipidemia (57.3 vs. 66.2%; *p* = 0.001), and chronic obstructive pulmonary disease (9.24 vs. 47.4%; *p* = 0.001). However, women had a higher prevalence of hypertension (93.0 vs. 89.2%; *p* = 0.007), higher rate of NYHA functional classes III–IV (38.5 vs. 31.5%; *p* = 0.003), and higher body mass index (30.9 ± 6.2 vs. 30.1 ± 5.8 kg/m^2^; *p* = 0.011) than men, whereas serum potassium (4.2 ± 0.43 vs. 4.3 ± 0.48 mmol/l; *p* = 0.010), blood urea nitrogen (16.2 ± 14.2 vs. 20.1 ± 14.3 mg/dl; *p* = 0.001), hemoglobin (12.6 ± 1.8 vs. 13.3 ± 2.4 g/dl; *p* = 0.001), and creatinine (1.0 ± 0.3 vs. 1.3 ± 0.31 mg/dl; *p* < 0.001) levels were lower in women than in men, and women had lower Kansas City Cardiomyopathy Questionnaire scores (54.1 ± 19.9 vs. 61.9 ± 21.3, *p* < 0.001) than men. Furthermore, elderly women with HFpEF had significantly higher left ventricular ejection fraction (63.3 ± 0.5 vs. 58.9 ± 0.6%, *p* = 0.001) and late mitral inflow velocity (81.5 ± 25.2 vs. 71.1 ± 26.8 cm/s, *p* < 0.001). Left ventricular filling pressure (E/Em) (12.5 ± 6.4 vs. 11.5 ± 5.9, *p* = 0.142) and E-wave deceleration time (210.4 ± 65.2 vs. 201.6 ± 67.1 s, *p* = 0.154) were higher in women than in men, but the differences in these parameters were not significant. Furthermore, plasma BNP [245 (148, 431) vs. 302 (165, 483) pg/ml, *p* = 0.039] was lower in women than in men, but there was no significant difference in the N-terminal pro-BNP [889 (485, 1,914) vs. 901 (532, 1,908) pg/ml, *p* = 0.787] level.

**Table 1:** Characteristics of the patients according to treatment arm.

	**All**			**Placebo arm**			**Spironolactone arm**		
	**Women (*n =* 898)**	**Men (*n =* 721)**	***p***	**Women (*n =* 445)**	**Men (*n =* 363)**	***p***	**Women (*n =* 453)**	**Men (*n =* 358)**	***p***
**Age, years**	77.0 ± 5.3	77.1 ± 5.1	0.458	76.6 ± 0.2	77 ± 0.3	0.116	77.4 ± 0.3	77.2 ± 0.3	0.621
**Region**
Americas, *n (%)*	533 (59.4)	498 (69.1)	<0.001	255 (56.0)	252 (69.4)	<0.001	278 (61.4)	246 (68.7)	0.03
Russia/Georgia, *n (%)*	365 (40.6)	223 (30.9)	<0.001	190 (42.7)	111 (30.6)	<0.001	175 (38.6)	112 (31.3)	0.03
**Atrial fibrillation**, ***n (%)***	371 (41.4)	341 (47.4)	0.016	188 (42.2)	164 (45.2)	0.399	183 (40.4)	177 (49.4)	0.01
**Coronary artery disease**									
Angina, *n (%)*	363 (40.4)	316 (43.9)	0.166	193 (43.4)	164 (45.2)	0.602	170 (37.5)	152 (42.5)	0.154
MI, *n (%)*	168 (18.7)	223 (30.9)	<0.001	86 (19.3)	120 (33.1)	<0.001	82 (18.1)	103 (28.8)	<0.001
CABG, *n (%)*	68 (7.6)	161 (22.3)	<0.001	41 (9.2)	89 (24.5)	<0.001	27 (6.0)	72 (20.1)	<0.001
PCI, *n (%)*	11 2(12.5)	136 (18.9)	<0.001	49 (11.0)	76 (20.9)	<0.001	63 (13.9)	60 (16.8)	0.261
**Hypertension**, ***n (%)***	835 (93.0)	643 (89.2)	0.007	417 (93.7)	326 (89.8)	0.042	418 (92.3)	317 (88.5)	0.071
**Diabetes mellitus**, ***n (%)***	252 (28.1)	229 (31.2)	0.105	117 (26.3)	123 (33.9)	0.019	135 (29.8)	106 (29.6)	0.953
**Dyslipidemia**, ***n (%)***	515 (57.3)	477 (66.2)	<0.001	258 (58.0)	250 (68.9)	0.001	257 (56.7)	227 (63.4)	0.054
**Tobacco use**, ***n (%)***	224 (24.8)	482 (66.9)	<0.001	114 (25.6)	236 (65.0)	<0.001	110 (24.3)	246 (67.8)	<0.001
**COPD**, ***n (%)***	83 (9.24)	118 (47.4)	<0.001	39 (8.6)	63 (17.6)	<0.001	44 (9.7)	55 (15.4)	0.015
**Heart rate, beats/min**	69.0 ± 10.4	67.2 ± 10.2	0.002	69.0 ± 0.5	67.5 ± 0.5	0.049	69.1 ± 0.5	66.8 ± 0.6	0.013
**SBP, mmHg**	130.5 ± 14.3	126.8 ± 13.5	<0.001	131.1 ± 0.7	126.6 ± 0.7	<0.001	129.9 ± 0.7	127.1 ± 0.7	0.009
**DBP, mmHg**	74.5 ± 11.1	71.9 ± 10.7	<0.001	74.5 ± 0.6	71.6 ± 0.6	<0.001	72.5 ± 0.5	72.1 ± 0.6	0.006
**Body mass index, kg/m** ^**2**^	30.9 ± 6.2	30.1 ± 5.8	0.011	31.0 ± 0.3	30.0 ± 0.3	0.024	30.7 ± 0.3	30.1 ± 0.3	0.164
**Serum potassium, mmol/L**	4.2 ± 0.4	4.3 ± 0.5	0.01	4.3 ± 0.0	4.29 ± 0.0	0.108	4.2 ± 0.2	4.3 ± 0.0	0.349
**Blood urea nitrogen, mg/dl**	16.2 ± 14.2	20.1 ± 14.3	<0.001	16.9 ± 0.7	18.8 ± 0.7	0.005	15.4 ± 0.6	21.4 ± 0.8	<0.001
**Creatinine, mg/dl**	1.0 ± 0.3	1.3 ± 0.3	<0.001	1.0 ± 0.0	1.2 ± 0.0	<0.001	1.0 ± 0.0	1.3 ± 0.0	<0.001
**Estimated GFR, ml/min/1.73 m** ^**2**^	60.8 ± 17.6	64.2 ± 17.5	<0.001	60.0 ± 0.8	65.1 ± 1.0	<0.001	61.5 ± 0.8	63.3 ± 0.9	0.073
**Hemoglobin, g/dL**	12.6 ± 1.8	13.3 ± 2.4	<0.001	12.8 ± 0.1	13.2 ± 0.1	<0.001	12.5 ± 0.1	13.4 ± 0.1	<0.001
**NYHA functional classes III–IV**, ***n (%)***	346 (38.5)	227 (31.5)	0.003	172 (38.7)	104 (28.7)	0.003	174 (38.4)	123 (34.4)	0.234
**LVEF (%)**	63.3 ± 0.5	58.9 ± 0.6	0.001	61.3 ± 0.7	59.3 ± 0.8	0.031	61.4 ± 0.6	58.5 ± 0.9	0.012
**E (cm/s)**	86.3 ± 26.9	86.7 ± 29.1	0.37	85.2 ± 24.8	88.6 ± 29.1	0.381	87.2 ± 28.7	84.6 ± 29.0	0.727
**A (cm/s)**	81.5 ± 25.2	71.1 ± 26.8	<0.001	83.9 ± 25.1	7.6 ± 26.0	0.001	79.3 ± 25.3	71.9 ± 27.9	0.11
**E/A**	1.1 ± 0.7	1.2 ± 0.7	0.108	1.1 ± 0.7	1.3 ± 0.7	0.064	1.2 ± 0.7	1.2 ± 0.7	0.712
**EDT (s)**	210.4 ± 65.2	201.6 ± 67.1	0.154	210 ± 73.5	198.5 ± 63.9	0.381	210.8 ± 57.3	205.1 ± 70.8	0.249
**E/Em septal**	16.5 ± 6.7	15.9 ± 7.7	0.262	16.3 ± 5.8	15.8 ± 7.5	0.345	16.6 ± 7.5	15.8 ± 8.0	0.513
**E/Em lateral**	12.5 ± 6.4	11.5 ± 5.9	0.142	12.6 ± 5.3	11.8 ± 6.3	0.149	12.4 ± 7.2	11.2 ± 5.5	0.436
**BNP (pg/ml)**	245 [148, 431]	302 [165, 483]	0.039	241.5 [147.5, 389.5]	307 [159, 454]	0.094	245 [151, 472]	284.5 [170,502]	0.234
**NT-proBNP (pg/ml)**	889 [485,1914]	901 [532, 1,908]	0.787	971 [560, 2,276]	904.5 [540.5, 2,025]	0.437	802 [435, 1,650]	901 [517, 1,790]	0.378
**KCCQ overall score**	54.1 ± 19.9	61.9 ± 21.3	<0.001	53.8 ± 0.9	61.8 ± 1.1	<0.001	54.3 ± 1.0	62.0 ± 1.2	<0.001
**PHQ-9 score**	5.3 ± 9.2	4.8 ± 8.8	0.065	5.5 ± 0.6	4.1 ± 0.8	0.078	5.0 ± 0.7	5.4 ± 0.3	0.377
**Any antihypertensive drugs**, ***n (%)***	892 (99.4)	713 (99.0)	0.334	441 (99.1)	360 (99.2)	0.825	451 (99.6)	353 (98.6)	0.144
ACEI or ARB, *n (%)*	730 (81.4)	568 (78.9)	0.21	365 (82.0)	282 (77.7)	0.126	365 (80.6)	286 (80.0)	0.807
Beta-blocker, *n (%)*	670 (74.7)	558 (77.5)	0.189	333 (74.8)	286 (78.8)	0.18	337 (74.4)	272 (76.0)	0.604
CCB, *n (%)*	356 (39.7)	256 (35.6)	0.089	186 (41.8)	125 (34.4)	0.033	170 (37.5)	131 (36.6)	0.784
Diuretic, *n (%)*	754 (84.1)	604 (83.9)	0.927	378 (84.9)	309 (85.1)	0.929	376 (83.0)	295 (82.4)	0.822
**Other antihypertensive drugs**, ***n (%)***	110 (12.3)	115 (16.0)	0.032	56 (12.6)	55 (15.2)	0.29	54 (11.9)	60 (16.8)	0.049
**Aspirin**, ***n (%)***	552 (61.5)	460 (63.9)	0.332	278 (62.5)	233 (64.2)	0.608	274 (60.5)	227 (63.4)	0.395
**Nitrate**, ***n (%)***	143 (15.9)	113 (15.7)	0.892	73 (16.4)	57 (15.7)	0.789	70 (15.5)	56 (15.6)	0.941
**Any hypoglycemic**, ***n (%)***	202 (22.5)	195 (27.1)	0.034	93 (20.9)	103 (28.4)	0.013	109 (24.1)	92 (25.7)	0.592
**Statin**, ***n (%)***	420 (46.8)	445 (61.8)	<0.001	204 (45.8)	220 (60.6)	<0.001	216 (47.7)	225 (62.8)	<0.001
**Warfarin**, ***n (%)***	231 (25.8)	229 (31.8)	0.007	116 (26.1)	115 (31.7)	0.078	115 (25.4)	114 (31.8)	0.043
**Other CV medication**, ***n (%)***	396 (44.1)	410 (56.9)	<0.001	192 (43.1)	207 (57.0)	<0.001	204 (45.0)	203 (56.7)	0.001
**Selective serotonin reuptake inhibitor**, ***n (%)***	78 (8.7)	50 (6.9)	0.195	39 (87.6)	26 (7.2)	0.406	39 (8.6)	24 (6.7)	0.314

*Values are mean ± SD or n (%)*.

*>CABG, coronary artery bypass graft; PCI, percutaneous coronary intervention; COPD, chronic obstructive pulmonary disease; GFR, glomerular filtration rate; HF, heart failure; LVEF, left ventricular ejection fraction; MI, myocardial infarction; SBP, systolic blood pressure; DBP, diastolic blood pressure; KCCQ, Kansas City Cardiomyopathy Questionnaire; NYHA, New York Heart Association; PHQ-9, Patient Health Questionnaire 9th edition; ACE, angiotensin-converting enzyme; ARB, angiotensin receptor blocker; CCB, calcium channel blocker; CV, cardiovascular; E, early mitral inflow velocity; A, late mitral inflow velocity; E/A, early to late mitral inflow velocity ratio; EDT, E wave deceleration time; E/Em, mitral inflow to mitral relaxation velocity ratio*.

Regarding the use of medications, no significant differences were noted for angiotensin-converting enzyme inhibitors, angiotensin receptor blockers, beta-blockers, calcium channel blockers, or diuretics between men and women. Men were significantly more likely to take statins, warfarin, any hypoglycemic drug, other antihypertensives, or CV medications. Moreover, in both the placebo and spironolactone arms, differences in the use of drugs between women and men were the same as the differences in the entire cohort.

### Differences in Outcomes Between Elderly Women and Men

**Figure 1:**
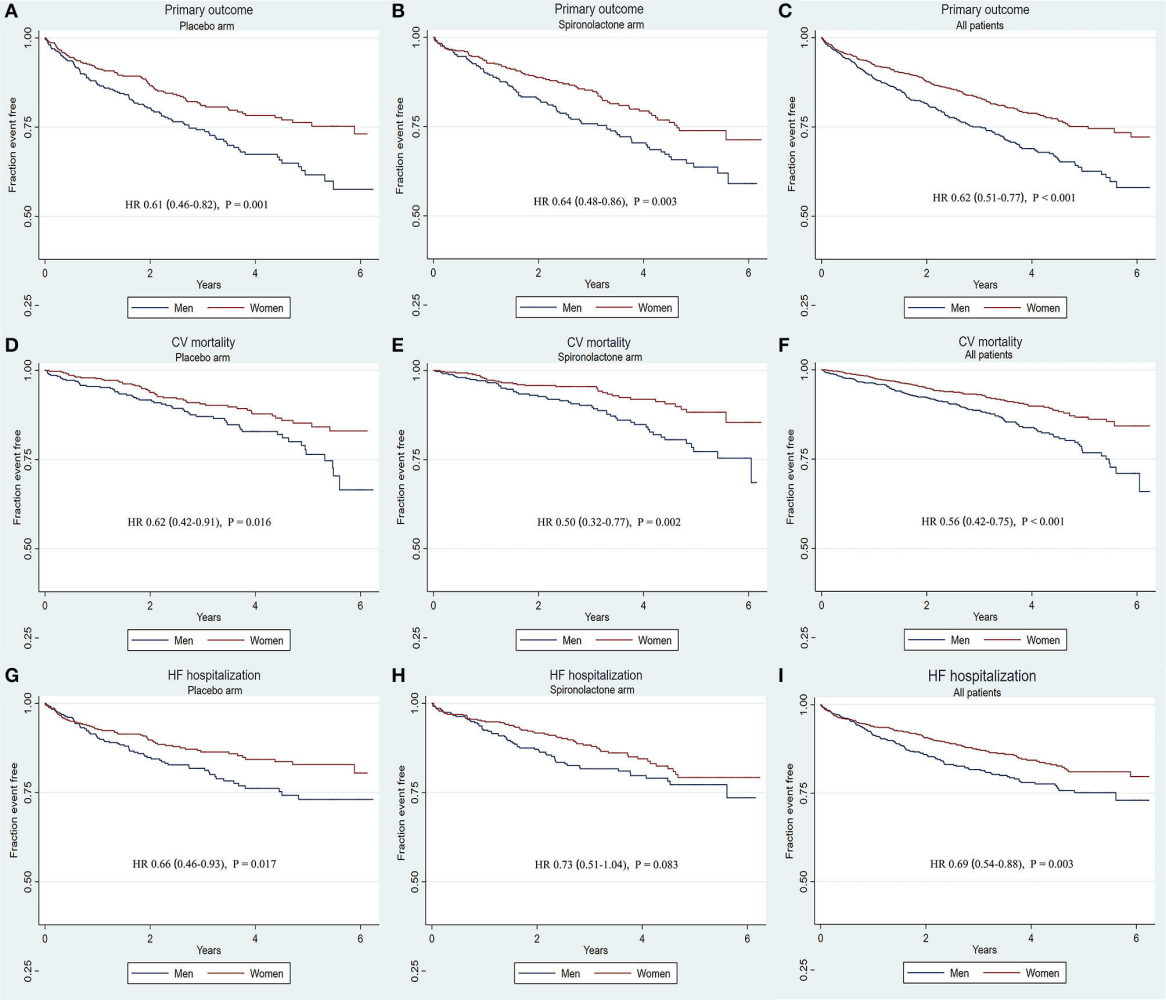
Kaplana–Meier survival curves for primary outcomes and components stratified by sex according to treatment. There was a significant association between sex and the primary outcome, CV mortality in either placebo arm or spironolactone arm. Women had a significantly lower rate of all the primary outcomes in all patients. **(A–C)** Primary outcome in placebo arm, spironolactone arm, all patients; **(D–F)** CV mortality in placebo arm, spironolactone arm, all patients; **(G–I)** HF-related hospitalization in placebo arm, spironolactone arm, all patients. CV, cardiovascular diseases; HF, heart failure; HR, hazard ratio.

**Figure 2:**
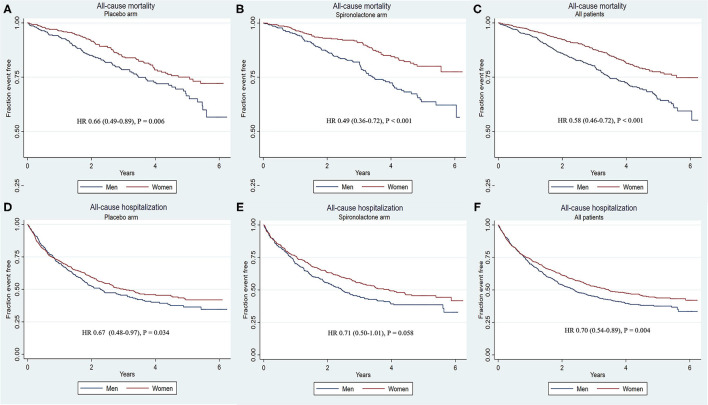
Kaplana–Meier survival curves for secondary outcomes and components stratified by sex according to treatment. Women were associated with a significantly reduced likelihood of all-cause mortality in the placebo arm, spironolactone arm, and all patients. No significant result was observed for all-cause hospitalization. **(A–C)** All-cause mortality in placebo arm, spironolactone arm, all patients; **(D–F)** All-cause hospitalization in placebo arm, spironolactone arm, all patients.

**Figure 3:**
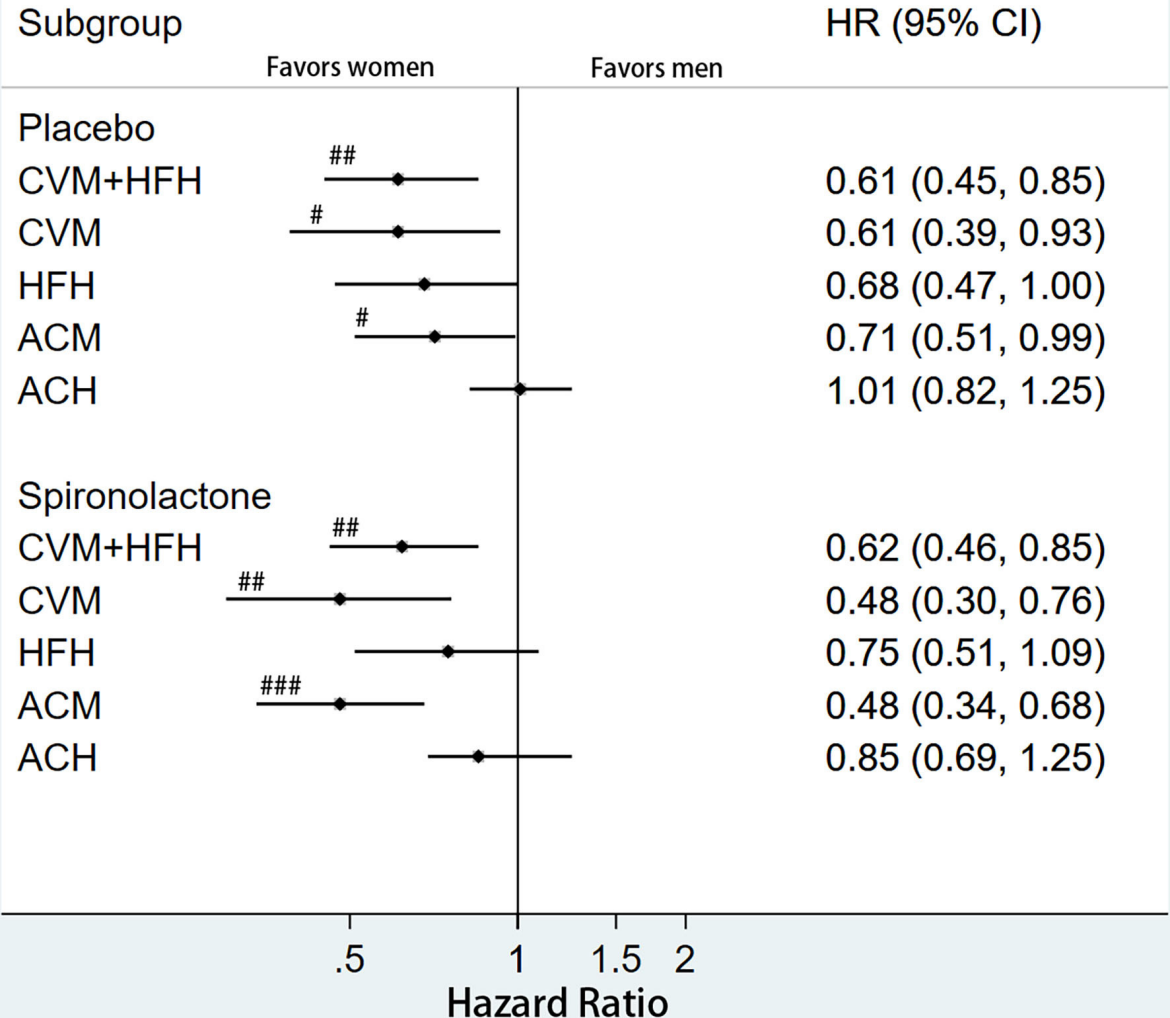
Multivariate hazard ratios for primary and secondary outcomes according to treatment arm and stratified by sex. ^#^*p* < 0.05, ^##^*p* < <0.01, ^###^*p* < 0.001. ACM, all-cause mortality; CVH, cardiovascular hospitalization; CVM, cardiovascular mortality; HFH, heart failure hospitalization; ACH, all-cause hospitalization.

The rates of primary and secondary outcomes according to sex between the placebo and spironolactone arms are summarized in Table 2. In the entire cohort, the rates of primary outcome (18.4 vs. 27.5%, *p* < 0.001), CV mortality (8.9 vs. 14.8%, *p* < 0.001), HF-related hospitalization (13.4 vs. 18.2%, *p* = 0.008), all-cause mortality (15.6 vs. 25.4%, *p* < 0.001), and all-cause hospitalization (47.0 vs. 52.3%, *p* = 0.012) were all significantly lower in women than in men. In the placebo arm, women had lower rates of composite primary outcomes (18.9 vs. 28.1%; *p* = 0.002), CV mortality (10.6 vs. 15.4%; *p* = 0.039), HF-related hospitalization (13.5 vs. 19.0%; *p* = 0.033), and all-cause mortality (31.5 vs. 50.4%; *p* < 0.001) than men. The rate of all-cause hospitalization was lower in women than in men (48.5 vs. 53.2%, *p* = 0.191), but the difference was not statistically significant. In patients treated with spironolactone, the rates of composite primary outcomes (17.9 vs. 26.8%; *p* = 0.002), CV mortality (7.3 vs. 14.2%; *p* = 0.001), all-cause mortality (13.0 vs. 25.7%; *p* < 0.001), and all-cause hospitalization (45.5 vs. 53.4%; *p* = 0.026) were significantly lower in women than in men. The HF-related hospitalization rate was lower in women than in men (13.2 vs. 17.3%, *p* = 0.107), but the difference was not statistically significant. Kaplan–Meier curves for primary and secondary outcomes stratified by sex are shown in Figures 1, 2. Sex-specific univariate analysis showed that women had lower rates of all outcomes in the entire cohort and in the placebo arm. In patients treated with spironolactone, no significant statistical differences were noted in HF-related hospitalization [hazard ratios (HR): 0.73; 95% confidence interval (CI): 0.51–1.04; *p* = 0.083] and all-cause hospitalization (HR: 0.71; 95% CI: 0.50–1.01; *p* = 0.058) between women and men, but primary outcome, CV mortality, and all-cause mortality were significantly lower in women than in men. Sex-specific multivariate HRs in the placebo and spironolactone arms for all outcomes adjusted for race, NYHA class, myocardial infarction, percutaneous coronary intervention, coronary artery bypass grafting, atrial fibrillation, hypertension, dyslipidemia, chronic obstructive pulmonary disease, systolic blood pressure, diastolic blood pressure, heart rate, body mass index, baseline estimated glomerular filtration rate, and baseline potassium levels are detailed in Supplementary Table 1 and Figure 3. In the entire cohort, the risk of primary outcomes, CV mortality, HF-related hospitalization, and all-cause mortality was significantly lower in women after adjusting for covariates. Also, both in the placebo and spironolactone arms, women were more likely to have significantly reduced risks of composite primary outcomes, CV mortality, and all-cause mortality compared with men.

**Table 2:** Differences in outcomes between women and men.

	**All**			**Placebo arm**			**Spironolactone arm**		
	**Women**	**Man**	***p***	**Women**	**Men**	***P***	**Women**	**Men**	***p***
	**(*n =* 898)**	**(*n =* 721)**		**(*n =* 445)**	**(*n =* 363)**		**(*n =* 453)**	**(*n =* 358)**	
**Primary outcome**, ***n (%)***	165 (18.4)	198 (27.5)	<0.001	84 (18.9)	102 (28.1)	0.002	81 (17.9)	96 (26.8)	0.002
CV mortality, *n (%)*	80 (8.9)	107 (14.8)	<0.001	47 (10.6)	56 (15.4)	0.039	33 (7.3)	51 (14.2)	0.001
HF hospitalization, *n (%)*	120 (13.4)	131 (18.2)	0.008	60 (13.5)	69 (19.0)	0.033	60 (13.2)	62 (17.3)	0.107
**All-cause mortality**, ***n (%)***	140 (15.6)	183 (25.4)	<0.001	140 (31.5)	183 (50.4)	<0.001	59 (13.0)	92 (25.7)	<0.001
**All-cause hospitalization**, ***n (%)***	422 (47.0)	384 (52.3)	0.012	216 (48.5)	193 (53.2)	0.191	206 (45.5)	191 (53.4)	0.026

*Values are n (%). Chi-square tests for women vs. men. Abbreviations as in Table 1*.

### Treatment Effect in Elderly Women and Men

Univariate HR for all outcomes is shown in Table 3. In women, primary outcomes occurred in 84 patients (10.4%) treated with placebo and in 81 patients (10.0%) treated with spironolactone (HR: 0.95; 95% CI: 0.70–1.29). The rates of CV mortality, HF-related hospitalization, all-cause mortality, and all-cause hospitalization were also lower in patients taking spironolactone, but the differences were not statistically significant (*p* > 0.05 for all outcomes). The effects of spironolactone treatment were similar among men.

**Table 3:** Univariate and multivariate hazard ratios and interaction terms between sex and treatment response for all outcomes.

**Outcome**	**Placebo**	**Spironolactone**	**Univariate**	**p**	**Multivariate**	***p***	***P* interaction**
	**(*n* = 808)**	**(*n* = 811)**	**HR (95% CI)**		**HR (95% CI)**		
**Primary outcome**							*0.781*
Women, *n* (%)	84 (10.4)	81 (10.0)	0.95 (0.70–1.29)	0.73	0.91 (0.67–1.25)	0.58	
Men, *n* (%)	102(12.6)	96 (11.8)	0.90 (0.68–1.19)	0.475	0.88 (0.66–1.17)	0.377	
**CV mortality**							0.525
Women, *n* (%)	47 (5.8)	33 (4.1)	0.70 (0.45–1.09)	0.117	0.66 (0.42–1.03)	0.068	
Men, *n* (%)	102 (12.6)	96 (11.8)	0.87 (0.60–1.28)	0.483	0.81 (0.55–1.20)	0.287	
**HF hospitalization**							0.699
Women, *n* (%)	60 (7.4)	60 (7.4)	0.98 (0.67–1.40)	0.921	0.93 (0.68–1.33)	0.683	
Men, *n* (%)	69 (8.5)	62 (7.6)	0.87 (0.62–1.23)	0.436	0.87 (0.61–1.24)	0.449	
**All-cause mortality**							0.19
Women, *n* (%)	81 (10.0)	59 (7.3)	0.73 (0.52–1.01)	0.062	0.68 (0.48–0.96)	0.028	
Men, *n* (%)	91 (11.3)	92 (11.3)	0.97 (0.73–1.30)	0.857	0.95 (0.70–1.28)	0.72	
**All-cause hospitalization**							0.325
Women, *n* (%)	216 (26.7)	206 (25.4)	0.89 (0.73–1.07)	0.212	0.84 (0.69–1.02)	0.086	
Men, *n* (%)	193 (23.9)	191 (23.6)	0.97 (0.80–1.19)	0.783	0.97 (0.79–1.19)	0.779	

*Values are n (%). Cox proportional hazards model to explore the associations between sex and the outcomes. Abbreviations as in Table 1*.

Multivariate HR and interaction terms between sex and treatment response are summarized in Supplementary Figure 1 and Table 3. No significant reduction was observed in the rate of primary outcomes associated with spironolactone in women (HR: 0.91; 95% CI: 0.67–1.25; *p* = 0.580) and men (HR: 0.88; 95% CI: 0.66–1.17; *p* = 0.377). The rates of CV mortality, HF-related hospitalization, and all-cause hospitalization were not significantly different between the placebo and spironolactone arms in both women and men (*p* > 0.05). Although women treated with spironolactone had a decreased rate of all-cause mortality (10.0 vs. 7.3%; HR: 0.68; 95% CI: 0.48–0.96, *p* = 0.028) compared with that noted in men treated with spironolactone, sex–treatment interactions were not significant (*p* for interaction = 0.190).

## Discussion

Patients older than 70 years from TOPCAT were included in the present study, and the following sex differences in baseline characteristics and outcomes were found: (1) elderly women with HFpEF had fewer comorbidities but worse cardiac function than men; (2) elderly women had a lower rate of primary outcomes, CV mortality, HF-related hospitalization, all-cause mortality, and all-cause hospitalization than men; and (3) although elderly women taking spironolactone had a lower rate of all-cause mortality than women taking placebo, there was no significant multivariate sex–treatment interaction.

### Sex Differences in Baseline Characteristics in Elderly Patients

Most of the HF cases happens in elderly patients, and more than half of patients hospitalized with HF are older than 75 years (14). It is reported that the prevalence of HF doubles for each decade of life. The prevalence is <1 and 10% for those younger than 40 years and older than 80 years, respectively (15). Moreover, a recent prospective study demonstrated that patients from different age groups have different clinical characteristics and outcomes (2). Previous HF trials examined sex differences for different age groups, such as age of ≥60 years in the I-PRESERVE study (8), age of ≥21 years in the DIG (Digitalis Investigation Group)—PEF (preserved ejection fraction) study (16), and age of ≥50 years in the TOPCAT—Americas study (17). Although significant sex differences in the baseline characteristics of patients with HFpEF have been reported, only a few studies have specifically focused on sex differences in elderly patients with HFpEF. Moreover, elderly HFpEF patients are underrepresented in large-scale randomized clinical trials. Thus, it is of great significance to emphasize sex difference in these elderly patients. In the present study, we focused on elderly patients from TOPCAT. We observed that hypertension, higher body mass index, and lower hemoglobin levels were more prevalent in elderly women. Men were more likely to be smokers and have coronary artery disease, atrial fibrillation, and chronic obstructive pulmonary disease. These findings were consistent with the data derived from the I-PRESERVE (8) and TOPCAT studies (17), and were also consistent with another meta-analysis (10) of the CHARM-Preserved (Candesartan in Heart Failure: Assessment of Reduction in Mortality and Morbidity), I-PRESERVE, and TOPCAT—Americas studies: female HFpEF patients are older and more likely to have obesity and hypertension but less likely to have coronary artery disease or atrial fibrillation. Pepine et al. reported in a recent study that the higher prevalence of obesity, hypertension, and other comorbidities in older women increases the prevalence of HFpEF in this group, which might explain that older women are more likely to develop HFpEF (18).

Furthermore, we observed that elderly women with HFpEF had worse NYHA functional classes and lower Kansas City Cardiomyopathy Questionnaire (19) scores (0–100, the higher scores, the fewer symptoms and physical limitations) than elderly men with HFpEF. The results suggested that elderly women were prone to have higher left ventricular ejection fraction, worse NYHA functional classes, more symptoms, and worse quality of life. We speculated that the worse NYHA functional classes and more symptoms in elderly women were attributable to impaired diastolic function. Women presented significantly higher late mitral inflow velocity (A) than men. Left ventricular filling pressure (E/Em) and E-wave deceleration time were numerically higher in women than in men but did not reach statistical significance, possibly due to the fact that many patients were without echo data. Consistent with previous studies, women with HFpEF had worse cardiac diastolic dysfunction than men. The possibility that diastolic cardiac function is more frequently abnormal in women is in agreement with the findings of previous studies (20–22): women with HFpEF have more prominent diastolic dysfunction than men with HFpEF. In contrast to the impaired diastolic function in women, lower BNP level was observed in elderly women with HFpEF. It was reported that women have higher plasma BNP level (23) in the general population but have lower BNP level in HFpEF due to the left ventricular concentric remodeling and hypertrophy among HFpEF patients (24). However, another study also reported that female HFpEF patients have a higher BNP level (21). Thus, the sex difference of BNP in HFpEF is still unclear and warrants further exploration.

Although women with HFpEF were more likely to have comorbid hypertension, no significant sex differences in the use of antihypertensive drugs, including angiotensin-converting enzyme inhibitors, angiotensin receptor blockers, beta-blockers, calcium channel blockers, or diuretics were observed. Based on previous studies (25–28), we speculated that these findings are attributed to different pharmacokinetics and pharmacodynamics: women taking angiotensin-converting enzyme inhibitors, angiotensin receptor blockers, and beta-blockers had higher plasma drug concentrations than men. Men were more likely to taking statins, warfarin, or hypoglycemic drugs than women, which could be explained by the fact that more men in our study had atrial fibrillation, diabetes, and dyslipidemia.

### Sex Differences in Outcomes in Elderly Patients

In the entire cohort, women not only had a lower rate of death including both CV mortality and all-cause mortality but also had a significantly lower rate of HF hospitalization. This result was inconsistent with a previous meta-analysis (10) involving 4,174 patients ≤70 years and 4,294 patients >70 years: women had better survival conditions than men but had similar rates of hospitalization. Another secondary analysis using data from the I-PRESERVE study (patients ≥60 years old) also indicated that women had significantly lower mortality rates and HF hospitalization than men (8). These results may suggest that the difference in the rate of HF-related hospitalization becomes more obvious with aging.

We also found that elderly women had significantly lower rates of composite primary outcome, CV mortality, and all-cause mortality than men both in the placebo arm and in the spironolactone arm. The rate of HF-related hospitalization in women were lower in women than in men even without a statistical significance. While in the study of Merrill (17), women and men present with similar clinical outcomes in the placebo arm, in the spironolactone arm, although women had better survival rate than men, the HF-related hospitalization was similar. The discrepant results might be caused by different age groups. Our study strictly selected patients ≥70 years, while the result of Merrill also included patients <70 years. The results might further confirm that men will have a higher rate of HF-related hospitalization with aging.

Interestingly, upon examining the differences in quality of life and outcomes between female and male HFpEF patients, we found that women, who had more symptoms and more physical limitations than men, had better outcomes. An observational study in 2018 has reported a similar result (21), such that quality of life was associated with HF severity and outcomes in men but not in women, whose quality of life was determined more by other unknown factors instead of HF itself. More evidence is needed to investigate the relationship between quality of life and outcomes in women with HFpEF. Combined with these studies, ways to improve quality of daily life are more pivotal for female patients, while exploring ways to improve outcomes for elderly male patients is more urgent even though they have fewer symptoms.

Apart from the fact of the different outcomes in elderly women and men with HFpEF, digging the causes is more important. Another secondary analysis from TOPCAT has reported that outcomes are influenced by key physiological factors that vary according to sex, such as ventricular vascular stiffening, which was the most significant determinant of outcomes in women, whereas in men, overall survival was influenced by heart rate and BNP levels (29). Thus, according to the varied determinants, controlling hypertension is key to improve outcomes in women while heart rate control may be beneficial to improve outcomes in men.

### Sex Differences of Spironolactone Treatment in Elderly Heart Failure With Preserved Ejection Fraction Patient

A previous meta-analysis (30) demonstrated that although mineralocorticoid receptor antagonists reduce morbidity and mortality rates in elderly patients with HFpEF more significantly. It also has the same effect on HFpEF, while in the current study, spironolactone therapy failed to reduce the rate of CV mortality or HF-related hospitalization in elderly patients with HFpEF. Moreover, after stratification by sex, there was no significant reduction in the rate of CV mortality and HF-related hospitalization associated with spironolactone in elderly women and men. This result is consistent with the findings of a previous study stating that the interaction between spironolactone and sex was not significant for CV mortality and HF-related hospitalization in the entire TOPCAT cohort (9) and the TOPCAT study restricted to the Americas (17). Although there was no significant sex–treatment interaction, spironolactone treatment had a significantly lower multivariate risk of the all-cause mortality in elderly women, suggesting a possible sex difference in spironolactone treatment concerning all-cause mortality.

### Limitations

First, all results are just hypotheses based on *post-hoc*, subgroup analysis selecting subjects older than 70 years from TOPCAT. Second, in order to make the sample size larger, we also included patients from Russia and Georgia, wherein the dose and treatments could vary between the Americas and other regions (Russia and Georgia) (31, 32), which might influence researchers to analyze treatment response. Finally, the *post-hoc* analysis was underpowered to assess sex differences in outcomes and response to treatment above the age of 75 years.

### Perspectives and Significance

This study showed that elderly women with HFpEF had worse clinical symptoms but better outcomes including both better survival and lower HF-related hospitalization than elderly men with HFpEF. Although the results were almost similar with studies that were not strictly limited to elderly patients, it did give an implication to us that men were more likely to have worse HF-related hospitalization with aging and provide stronger evidence for gender differences in HFpEF. Exploring the in-depth mechanism of HFpEF prognostic differences caused by sex differences is emergent and may help discover new targets for HFpEF treatment according to sex in the future.

In conclusion, our results showed that elderly women with HFpEF had fewer comorbidities but were more likely to have worse NYHA functional classes and worse quality of life than men. Importantly, elderly women not only had a better survival but also a lower rate of HF-related hospitalization than elderly men. It is worth noting that spironolactone is possibly associated with a reduced rate of all-cause mortality in elderly women.

## Data Availability Statement

Publicly available datasets were analyzed in this study. This data can be found at: the datasets used or analyzed during the current study are available from the National Heart, Lung, and Blood Institute's Biological Specimen and Data Repository Information Coordinating Center (BioLINCC, Calverton, Maryland). NCT00094302 (TOPCAT). https://www.clinicaltrials.gov/ct2/show/NCT00094302.

## Ethics Statement

Ethical review and approval was not required for the study on human participants in accordance with the local legislation and institutional requirements. Written informed consent for participation was not required for this study in accordance with the national legislation and the institutional requirements.

## Author Contributions

The work presented here was carried out in collaboration with all authors. SZ defined the study theme and methods. JS and ST collected clinical data, analyzed the data, interpreted the results, and wrote the paper. YG, LT, HY, XL, ZX, and LF are the attending doctor responsible for reviewing and giving suggestions to the manuscript. All authors contributed to the article and approved the submitted version.

## Funding

This research was partly supported by the Natural Science Foundation of China (NSFC) Projects 81670269 (to SZ) and 81801394 (to ST) and Natural Science Foundation of Hunan Province 2019JJ50878 (to ST).

## Conflict of Interest

The authors declare that the research was conducted in the absence of any commercial or financial relationships that could be construed as a potential conflict of interest.

## Publisher's Note

All claims expressed in this article are solely those of the authors and do not necessarily represent those of their affiliated organizations, or those of the publisher, the editors and the reviewers. Any product that may be evaluated in this article, or claim that may be made by its manufacturer, is not guaranteed or endorsed by the publisher.

## Abbreviations

CV: cardiovascular
HF: heart failure
NYHA: New York Heart Association
HFpEF: heart failure with preserved ejection fraction
BNP: brain natriuretic peptide.
