# Supplementary material for: Sex Differences in Characteristics and Outcomes in Elderly Heart Failure Patients With Preserved Ejection Fraction: A Post-hoc Analysis From TOPCAT
Source: Front Cardiovasc Med. 2021 Oct 4;8:721850. doi: 10.3389/fcvm.2021.721850 (PMC8520937; doi:10.3389/fcvm.2021.721850)
Supplement: Supplementary file 1 [file Data_Sheet_1.docx]

**Supplemental Table 1 – Multivariate hazard ratios and interaction terms between sex and treatment response for outcomes.**

| Outcome  (Women vs Men) | All | | Placebo arm | | | Spironolactone arm | | |
| --- | --- | --- | --- | --- | --- | --- | --- | --- |
|  | HR (95% CI) | *P* | | HR (95% CI) | *P* | | HR (95% CI) | *P* |
| Primary outcome | 0.60(0.48-0.76) | <0.001 | | 0.61(0.45-0.85) | 0.003 | | 0.62(0.46-0.85) | 0.003 |
| CV mortality | 0.53(0.40-0.73) | <0.001 | | 0.61(0.39-0.93) | 0.023 | | 0.48(0.30-0.76) | 0.002 |
| HF hospitalization | 0.71(0.55-0.93) | 0.013 | | 0.68(0.47-1.00) | 0.052 | | 0.75(0.51-1.09) | 0.129 |
| All-cause mortality | 0.59(0.47-0.75) | <0.001 | | 0.71(0.51-0.99) | 0.046 | | 0.48(0.34-0.68) | <0.001 |
| All-cause hospitalization | 0.93(0.80-1.08) | 0.356 | | 1.01(0.82-1.25) | 0.905 | | 0.85(0.69-1.05) | 0.138 |

**Supplemental Fig1**


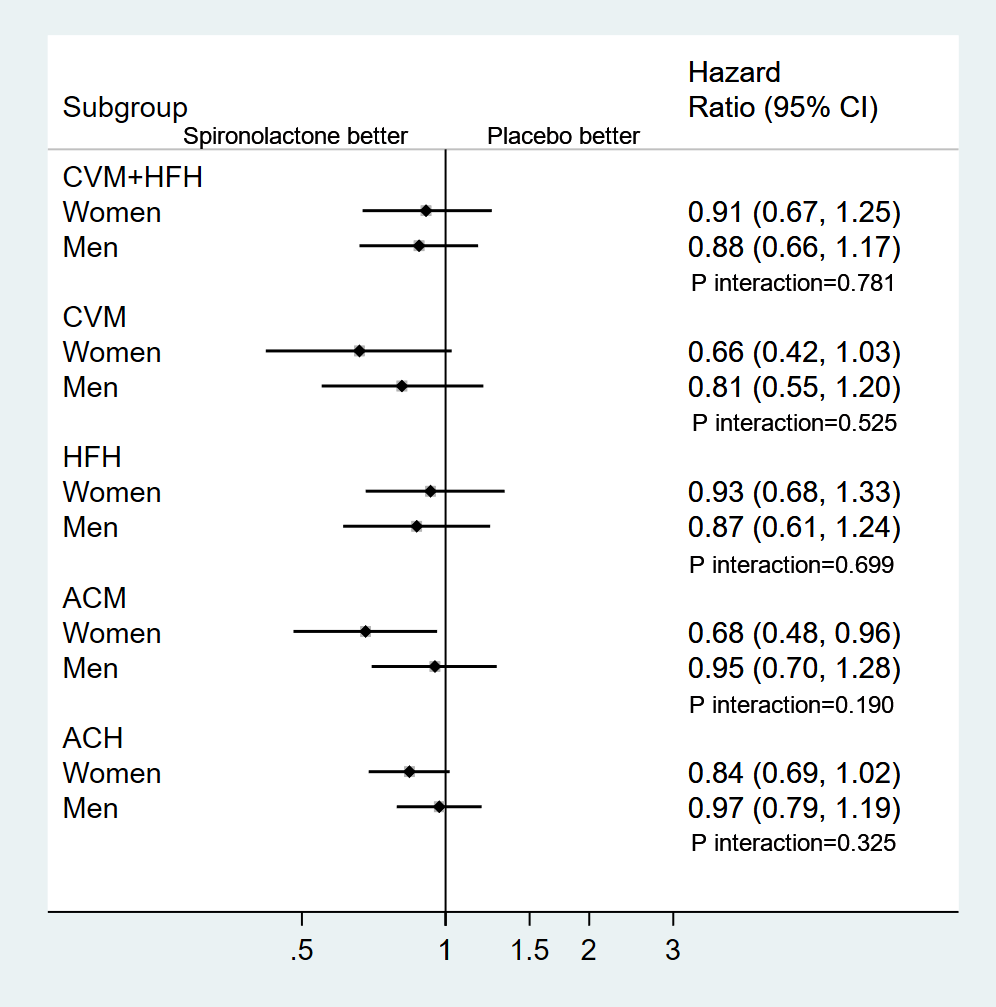


**Supplemental Fig1** Multivariate hazard ratios for outcomes according to sex and stratified by treatment. Spironolactone was associated with a reduced likelihood of all-cause mortality in women but not in men, but there was non-significant sex-treatment interaction. There were no other significant associations between spironolactone and outcomes. ACM=all-cause mortality; ACH=all-cause hospitalization; CVH=cardiovascular hospitalization; CVM= cardiovascular mortality; HFH= heart failure hospitalization. #: p <0.05
